# Māori and Linked Administrative Data: A Critical Review of the Literature and Suggestions to Realise Māori Data Aspirations.

**DOI:** 10.23889/ijpds.v7i3.1793

**Published:** 2022-08-25

**Authors:** Lara Greaves, Cinnamon Lindsay, Eileen Li, Emerald Muriwai, Andrew Sporle

**Affiliations:** 1 University of Auckland; 2 iNZight Analytics

Linked data presents different social and ethical issues for different contexts and communities. The Statistics New Zealand Integrated Data Infrastructure (IDI) is a collection of de-identified whole-population administrative datasets that researchers are increasingly using to answer pressing social and policy research questions. Our work seeks to provide an overview of the IDI, associated issues for Māori (the Indigenous peoples of New Zealand), and steps to realise Māori data aspirations. In this paper, we first introduce the IDI including what it is and how it developed. We then move to an overview of Māori Data Sovereignty. Our paper then turns to examples of organisations, agreements, and frameworks which seek to make the IDI and data better for Māori communities. We then discuss the main issues with the IDI for Māori including technical issues, deficit-framed work, involvement from communities, consent, social license, further data linkage, and barriers to access for Māori. We finish with a set of recommendations around how to improve the IDI for Māori, making sure that Māori can get the most out of administrative data for our communities. These include the need to build data researcher capacity and capability for Māori, Māori data co-governance and accountability, reducing practical and skill barriers for access by Māori and Māori organisations, providing robust, consistent and transparent practice exemplars for best practice, and potentially even abolishing the IDI and starting again. These issues are being worked through via Indigenous engagement and co-governance processes that could provide useful exemplars for Indigenous and community engagement with linked data resources.

